# Ventriculo‐arterial coupling in children with Still's murmur

**DOI:** 10.14814/phy2.12041

**Published:** 2014-07-03

**Authors:** Juliane Engel, Sigrid Baumgartner, Silvia Novak, Christoph Male, Ulrike Salzer‐Muhar

**Affiliations:** 1 Division of Pediatric Cardiology, Department of Pediatrics and Adolescent Medicine, Medical University of Vienna, Vienna, Austria; 2 Division of Neonatology, Intensive Care Medicine and Neuropediatrics, Department of Pediatrics and Adolescent Medicine, Medical University of Vienna, Vienna, Austria

**Keywords:** Children, echocardiography, Still's murmur, ventriculo‐arterial coupling

## Abstract

Still's murmur is the most common innocent heart murmur in children and considered flow related; however, so far the cause of the murmur has not yet been fully explained. Assessment of the hemodynamic ventriculo‐arterial interaction and the proportional anatomical dimensions of the left ventricle and the aortic root were the objective for this study. This case–control study was conducted at the Division of Pediatric Cardiology, Vienna Medical University, including healthy children with and without Still's murmur. To assess ventriculo‐arterial interaction, the model of ventriculo‐arterial coupling (VAC) was applied. The model describes the interaction between the left ventricle (left ventricular contractility, E_LV_) and the arterial system (effective arterial elastance, E_A_) by the VAC ratio E_A_/E_LV_. The parameters E_A_ and E_LV_ can be derived from M‐mode echocardiography thereby allowing a noninvasive pressure–volume analysis. Outcomes comprised VAC ratio and diameters of both the aortic root (AOD) and the left ventricle in end diastole (LVED) and end systole (LVES) as well as their relative proportions, ejection fraction (EF), stroke volume (SV), blood pressure (BP), and heart rate (HR). Forty‐three healthy children with Still's murmur (mean age 5.2 years) and 42 healthy children without murmur (mean age 5.8 years) participated in this study. Children with Still's murmur had a significantly lower VAC ratio E_A_/E_LV_ (0.5 ± 0.13 vs. 0.59 ± 0.15; *P* < 0.005), a significantly higher EF% (67.1 ± 5.8 vs. 63.3 ± 5.6; *P* < 0.005, *P* < 0.01), and a larger SV per kg bodyweight (1.84 ± 0.33 vs. 1.68 ± 0.38; *P* < 0.05) than controls. BP, HR, and diameters of AOD, LVED, and LVES as well as their relative anatomic proportions did not differ between children with Still's murmur and controls. Still's murmur seems to be generated by a subtle alteration in ventriculo‐arterial coupling in healthy children. This result can be translated to parents, as they may be informed that their child's innocent murmur is caused by a more “lively interplay between the heart and the aorta.”

## Abstract (precis)

So far, the physiology of Still's murmur has not yet been defined. This study is the first to describe a significant difference in ventriculo‐arterial coupling in healthy children with and without Still's murmur.

## Introduction

Innocent heart murmurs are a common phenomenon in pediatric cardiology, occurring in most children at some point during childhood and having been known for well over 100 years (Biancaniello 2005). By definition, they are “normal” or “physiological” heart murmurs not related to congenital or acquired heart disease. Using the term “innocent” is one way of emphasizing their harmlessness and usually helps to reassure patients and parents (Biancaniello 2004). Innocent heart murmurs present a soft and musical sound and are considered flow related (Gardiner and Joffe 1991; Celebi and Onat 2006).

Classic innocent murmurs are the pulmonary flow murmurs in adolescents, the systemic flow murmurs (“carotic bruits”), the cervical venous hum (Biancaniello 2005), and most common, Still's murmur which is in the focus of this study (Still 1909).

Still's murmur has its peak prevalence in children aged 3–6 years, with a fast decrease in adolescence. It is described as a grade 1‐2/6 low‐frequency vibratory or musical murmur with a punctum maximum in the third to fourth left intercostal space/at the lower left sternal border with little to no transmission. The murmur changes noticeably with any change in position and presents loudest in supine position (Biancaniello 2005).

So far, there is no definite explanation for Still's murmur. At the same time this murmur is not only the most common reason to refer a child to echocardiography but also the cause of considerable doctoral uncertainty and parental anxiety.

One common explanation willingly accepted by both parents and doctors focuses on the high occurrence of left ventricular false tendons in children with Still's murmur, which were thought to be responsible for the soft vibrating sound that resembles a swinging violin string. However, the high prevalence of both children with left ventricular false tendons but no murmur and children with Still's murmur but no sign of left ventricular false tendons does not support this explanation (Kervancioğlu et al. 2003). Early studies in children and young adults suggested that the origin of Still's murmur is related to a small ascending aortic diameter with concomitant high aortic blood flow velocity (Schwartz et al. 1986). Schoolchildren presenting with a vibratory innocent heart murmur were shown to have a significantly lower heart rate and a significantly smaller diameter of the ascending aorta than matched controls (Van Oort et al. 1994), whereas others while confirming relative bradycardia and higher aortic flow volume could not find any correlation between aortic size and Still's murmur (Gardiner and Joffe 1991).

The discrepancy between the frequency of Still's murmur in everyday pediatric cardiology and the remaining uncertainties regarding its explanation was the rationale to study the physiology of Still's murmur using a different approach. Expanding on the observation that children with Still's murmur have significantly smaller aortic roots than those without (Van Oort et al. 1994), we decided to focus on the left ventricle and the aorta. Our aim was to study the hemodynamic interaction between the left ventricle and the aorta and the proportional anatomical dimensions of the left ventricle and the aortic root. We hypothesized that Still's murmur is either linked to the hemodynamic interaction between the left ventricle and the aorta, respectively, the arterial system, or to a disproportion between left ventricular and aortic root dimensions.

To study the hemodynamic interaction between the left ventricle and the aorta, we decided to apply the hemodynamic model of ventriculo‐arterial coupling (VAC) that has been used in studies on hypertension, heart failure, and other cardiovascular diseases (Borlaug and Kass 2008; Chantler and Lakatta 2012; Kuznetsova et al. 2012) since its introduction about three decades ago (Sunagawa 1983). The VAC model describes the heart – the pump – on the basis of the pressure–volume relation and the arterial system – the load – on the basis of the arterial windkessel (Westerhof et al. 2005). The VAC ratio E_A_/E_LV_ is a measure of the pressure–volume relationship at the threshold between the left ventricular and the arterial system, the respective parameters are effective arterial elastance (E_A_) and left ventricular end‐systolic contractility (E_LV_; Chantler et al. 2008). The parameters E_A_ and E_LV_ can be derived from echocardiography thereby allowing a noninvasive pressure–volume analysis.

To study the anatomic dimensions we decided to base our work on traditional M‐mode echocardiography.

Thus, the primary objective of this study was to analyze the VAC ratio E_A_/E_LV_ in healthy children with and without Still's murmur. Secondary objectives were to analyze left ventricular and aortic root diameters, their respective proportional dimensions, as well as blood pressure, heart rate, and other hemodynamic variables such as ejection fraction, stroke volume and cardiac output.

## Methods

### Setting and participants

The study was designed as a case–control study in children aged 2–10 years with and without Still's murmur and no previously known heart disease at the Division of Pediatric Cardiology at the Medical University of Vienna. The children had been referred for clinical and echocardiographic evaluation. They had been clinically examined by two senior pediatric cardiologists (S. N., U. S.‐M.) with profound skills in auscultation enabling them to give an objective detailed description of any murmur. Both doctors were well acquainted with the clinical characteristics of Still's murmur (Biancaniello 2005).

The evaluation included an electrocardiogram, blood pressure measurements, and a detailed echocardiography including M‐mode, 2D, and Doppler echocardiography according to existing standards to rule out even subtle anatomic findings. All children had been diagnosed as having normal cardiac morphology and function and normal Doppler echocardiographic findings without any turbulences within their heart and the great arteries, respectively.

### Data generation

#### Clinical data

In our institution, both standardized electronic clinical examination forms (including among many other items the documentation of the physical state (normal, asthenic, obese), the inspection of the chest (pectus carinatum/excavatum, scoliosis, precordial bulge), and the detailed description of any encountered murmur (punctum maximum, grade, phase, form, quality and transmission) and standardized electronic medical report forms are used and stored immediately digitally in the Patient Database of the Division of Pediatric Cardiology. Blood pressures are routinely measured by the oscillometric method, using a device from the GE‐series DINAMAP.

#### Echocardiographic data

Echocardiography including 2D imaging, M‐mode, and Doppler is performed as described in the *Guidelines of the American Society of Echocardiography* (Lai et al. 2006) on a GE Vivid 7 ultrasound machine, using either a 5‐ or 3‐MHz transducer. All the obtained images and standardized reports are transferred online to the ECHO‐Database at the time of the examination.

### Data source and inclusion criteria

Clinical examination data and M‐mode echocardiographic data were retrieved from the Patient Database and the ECHO Database, respectively. Data were anonymized for statistics.

### Study population

The inclusion criterion for cases was defined as

- Presence of a protomeso‐systolic musical murmur grade 1 or 2 with a punctum maximum at the left sternal border.
  Inclusion criteria for both cases and controls were defined as

- Absence of any illness at time of evaluation.
- Absence of history of any congenital or acquired structural cardiac disease.
- Absence of history of any extracardiac diagnosis, symptoms, or medication to be expected to have any effect on cardiac morphology or hemodynamics.
- Echocardiographic examination at age 2–10 years in the study period from January 2008 to March 2012 by those two pediatric cardiologists mentioned above.
  The study protocol was approved by the Ethics Committee, Medical University of Vienna (registration number 621/2010).

### Data and definitions

#### Clinical variables

Anthropometic variables included age, height, and weight, the body surface area (BSA) was calculated according to Haycock and Schwartz (1978) and Sluysmans and Colan (2005). Hemodynamic variables comprised systolic (SBP) and diastolic blood pressure (DBP) and heart rate (HR). Mean arterial pressure (MAP) and pulse pressure (PP) were calculated as follows: MAP = DPB + (SBP + DBP)/3 (Schumacher et al. 1978) and PP = SBP − DBP, respectively (Benetos et al. 1997).

#### Variables derived from M‐mode echocardiography

Measurements performed at the level of the left ventricle and the aortic root from the parasternal long‐axis view comprised left ventricular end‐diastolic (LVED) and end‐systolic (LVES) and aortic root (AOD) diameters. All diameters were also expressed in both their ratio to body surface area and regarding their relative proportions (AOD/LVED and AOD/LVES).

Left ventricular fractional shortening (FS%) was automatically calculated by the GE technology according to the following formula: FS = LVED − LVES/LVED (normal range 30–40%) (Lang et al. 2006; Sahn et al. 1978). Calculations of end‐systolic (ESV) and end‐diastolic left ventricular volumes (EDV), stroke volume (SV), and, in consequence, ejection fraction (EF%), from M‐mode measurements were generated based on the formula by Teichholz et al. (1976). Mean arterial pressure was used for determining arterial resistance (R), as MAP = R × CO and CO = SV × HR (Safar 1989), where CO is cardiac output in liters per minute.

#### Calculation of ventriculo‐arterial coupling and its parameters

The VAC ratio E_A_/E_LV_ was calculated as follows. End‐systolic pressure (ESP) being the parameter for the calculation of both E_A_ and E_LV_ can be calculated from SPB and DPB values by the following two formulae:

ESP_1_ = ([2 × SBP] + DBP)/3 or ESP_2_ = 0.9 × SBP (Kuznetsova et al. 2012). In the present study both formulae were used.

E_A_ is considered a measure of the net arterial load which is imposed on the left ventricle (Sunagawa 1983). It can be approximated noninvasively by end‐systolic pressure over stroke volume (ESP/SV; Kelly et al. 1992) which was done in this study using both ESP 1 and ESP 2 for calculating E_A1_ = ESP_1_/SV and E_A2_ = ESP_2_/SV, respectively.

E_LV_ is considered a load‐independent measure of left ventricular contractility and left ventricular performance. Noninvasive approximation of E_LV_ is possible by calculating ESP over end‐systolic volume (ESV; Chantler and Lakatta 2012) which was done in the present study using both ESP_1_ and ESP_2_ for calculating E_LV1_ = ESP_1_/ESV and E_LV2_ = ESP_2_/ESV, respectively.

In the present study calculations of E_A_ and E_LV_ were based on the common parameter of ESP and the volume measurements of SV and ESV, respectively. All calculations were performed using both ESP_1_ and ESP_2_ as described above.

Thus, the VAC ratio E_A_/E_LV_ was finally calculated as follows:

VAC = ESV/SV or VAC = E_A1_/E_LV1_ or VAC = E_A2_/E_LV2_

### Statistical analysis

All data were analyzed using IBM SPSS 19 (IBM Corp, Released 2010, IBM SPSS Statistics for Windows, Version 19.0, Armonk, NY). After ascertaining normal distribution of the relevant variables with the Kolmogorow–Smirnow test, the *t*‐test for independent samples was applied. The level of significance was defined as *P* < 0.05. All analyses were performed for the total population of cases and controls aged 2–10 years. As Still's murmur is known to disappear with growth (Biancaniello 2005), all analyses were also performed for subgroups according to age, dividing cases and controls into groups of children aged 2–5.9 years (group 1) and 6–10 years (group 2), respectively.

## Results

### Study population

Forty‐three children with Still's murmur (cases) and 42 controls, aged from 2 to 10 years met the inclusion criteria and formed the total study population. Subgroup 1 comprised 30 cases and 25 controls (mean age 4.1 ± 0.9 years vs. 4.0 ± 1.4 years), while subgroup 2 comprised 13 cases and 17 controls (mean age 7.7 ± 1.3 vs. 8.8 ± 1.1 years).

### Clinical variables

No significant differences between cases and controls could be found for any of the clinical variables in neither the total study population (Table 1) nor the subgroups.

**Table 1.** Clinical variables of children with Still's murmur (M) and controls (C).

	M (*N* = 43)	C (*N* = 42)	*P*
Age (y)	5.2 ± 2.0	5.8 ± 2.5	0.2
Height (cm)	110.3 ± 13.4	115.0 ± 15.6	0.1
BSA (m²)	0.77 ± 0.17	0.82 ± 0.18	0.3
SBP (mmHg)	101.1 ± 12.7	100.5 ± 10.3	0.8
DBP (mmHg)	56.7 ± 8.1	58.6 ± 10.9	0.37
PP (mmHg)	43.8 ± 10.3	42.6 ± 13.1	0.63
HR (bpm)	96.0 ± 18.2	101.3 ± 19.6	0.2
MAP (mmHg)	71.3 ± 7.5	72.8 ± 9.8	0.44

*P*, level of significance defined as <0.05. BSA, body surface area; SBP, systolic blood pressure; DBP, diastolic blood pressure; PP, pulse pressure; HR, heart rate; MAP, mean arterial pressure.

### Variables derived from M‐mode echocardiography

There were no significant differences in aortic root and left ventricular dimensions as well as in their proportions between cases and controls, neither in the total study population nor in the subgroups. Ejection fraction (*P* < 0.005) and SV/kg bodyweight (*P* < 0.05) were significantly higher in cases than in controls in the total study population but not in the subgroups (Table 2).

**Table 2.** Variables derived from M‐mode echocardiography.

	M (*N* = 43)	C (*N* = 42)	*P*
AOD/BSA (mm/m²)	25.4 ± 3.8	25.3 ± 4.0	0.83
LVED/BSA (mm/m²)	46.7 ± 6.1	44.8 ± 7.2	0.2
LVES/BSA (mm/m²)	29.7 ± 4.7	29.6 ± 5.1	0.9
EF (%)	67.1 ± 5.8	63.3 ± 5.6	<0.005
SV/kg (mL/kg)	1.84 ± 0.33	1.68 ± 0.38	<0.05
CO (L/min)	3.23 ± 0.81	3.34 ± 0.88	0.5
R (MAP/CO)	0.023 ± 0.005	0.023 ± 0.006	0.88
AOD/LVED (mm)	0.55 ± 0.06	0.56 ± 0.05	0.2
AOD/LVES (mm)	0.87 ± 0.11	0.85 ± 0.09	0.6

Children with murmur (M), controls (C). *P*, level of significance defined as <0.05. AOD, aortic root diameter; BSA body surface area; LVED, left ventricular end‐diastolic diameter; LVES, left ventricular end‐systolic diameter; EF, ejection fraction; SV/kg, stroke volume per kg bodyweight; CO, cardiac output; R, arterial resistance (*P* mean/CO).

### Ventriculo‐arterial coupling

**Figure 1.**
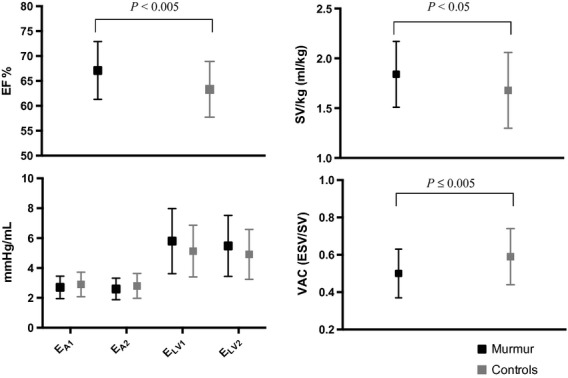
Calculated results. Set of diagrams showing the results for ejection fraction (EF%), stroke volume (mL/kg), effective arterial elastance (E_A1_, E_A2_) and left ventricular end‐systolic contractility (E_LV1_, E_LV2_) and ventriculo‐arterial coupling (VAC ratio = end‐systolic left ventricular volume [ESV] over end‐diastolic left ventricular volume [EDV]) in children with Still's murmur (M, black) and children without murmur (C, gray). The detailed calculation of E_A1_, E_A2_, E_LV1_, and E_LV2_ are described in the Methods section.

The VAC ratio (ESV/SV) was significantly lower in cases than in controls (*P* < 0.005) in the total study population (Table 3, Fig. 1). The same was true for subgroup 1 (0.50 ± 0.11 in cases vs. 0.6 ± 0.16 in controls; *P* < 0.05) but not for subgroup 2 (0.5 ± 0.17 in cases vs. 0.57 ± 0.13 in controls).

**Table 3.** Ventriculo‐arterial coupling and its parameters.

	M (*N* = 43)	C (*N* = 42)	*P*
ESP_1_	90.5 ± 9.2	91.0 ± 11.4	0.8
ESP_2_	85.9 ± 8.3	87.0 ± 10.5	0.61
E_A1_	2.7 ± 0.75	2.9 ± 0.82	0.39
E_A2_	2.6 ± 0.72	2.8 ± 0.83	0.33
E_LV1_	5.8 ± 2.18	5.13 ± 1.73	0.14
E_LV2_	5.48 ± 2.04	4.91 ± 1.67	0.17
ESV/SV	0.50 ± 0.13	0.59 ± 0.15	0.005
E_A1_/E_LV1_	0.50 ± 0.13	0.59 ± 0.15	0.005
E_A2_/E_LV2_	0.50 ± 0.13	0.59 ± 0.15	0.005

Children with murmur (M), controls (C). *P*, level of significance defined as <0.05. ESP_1_, end‐systolic pressure; ESP_2_, end‐systolic pressure 2; E_A_, effective arterial elastance; E_LV_, left ventricular contractility; ESV/SV, ventricular‐arterial coupling (end‐systolic volume over stroke volume); E_A_/E_LV_, ventricular‐arterial coupling (effective arterial elastance over left ventricular contractility).

Although E_A_ and E_LV_ did not differ significantly between cases and controls, there was a trend toward a slightly lower E_A_ and a higher E_LV_ in cases compared to controls in both the total study population (Fig. 1) and subgroup 1.

## Discussion

The discrepancy between the frequency of Still's murmur in everyday pediatric cardiology and the remaining uncertainties regarding its explanation was the rationale to study the physiology of Still's murmur. To contribute to the explanation of Still's murmur, we decided to focus on the left ventricle and the aorta, namely on the hemodynamic ventriculo‐arterial interaction and the proportional anatomical dimensions of the left ventricle and the aortic root.

When we applied the hemodynamic model of ventriculo‐arterial coupling (Sunagawa 1983; Chantler et al. 2008; Chantler and Lakatta 2012) in healthy 2‐ to 10‐year‐old children with and without Still's murmur we found that the VAC ratio was lower in children with Still's murmur than in those without.

As the VAC ratio in itself does not provide sufficient explanation for a murmur its parameters E_A_ and E_LV_ were examined more closely. They indicated a trend toward a lower arterial elastance and a higher left ventricular contractility in children with Still's murmur. In combination, both parameters contributed to the significant difference in VAC.

As Still's murmur is known to disappear with growth (Biancaniello 2005), a subgroup analysis according to age was performed. In the group of young children with Still's murmur, albeit not in the older ones the VAC ratio was also found to be significantly lower than in controls. Likewise, the abovementioned trend toward a slightly lower arterial elastance and a higher left ventricular contractility was more pronounced in the group of young children presenting with Still's murmur. These findings are in line with the clinical knowledge that Still's murmur is an age‐related clinical phenomenon.

Both ejection fraction and stroke volume per kg bodyweight were significantly higher in children with Still's murmur. Stroke volume is one of the parameters in the formula for calculating the VAC ratio what may partly explain the finding described above. It seems that in children with Still's murmur a significantly larger volume is transitioned into a less compliant arterial system.

In this regard, blood pressure is of interest as it was used in our study for the calculation of end systolic left ventricular pressure. As ESP was not found to be different in children with and without murmur it is unlikely to be the factor responsible for the significant difference regarding the VAC ratio. The same was true for both mean pressure and pulse pressure, the latter being indicative for peripheral arterial stiffness and known as a reliable predictor of cardiovascular disease (Chantler et al. 2008).

**Figure 2.**
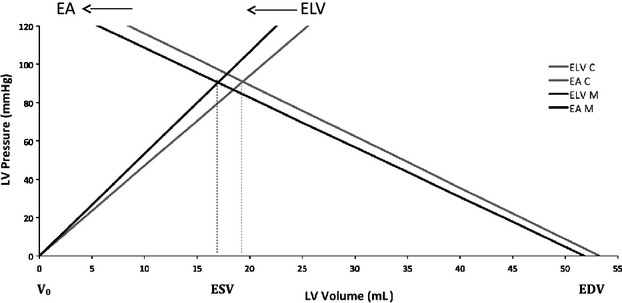
Pressure–volume relationships. Comparing the slope of the end‐systolic pressure–volume relationships and the slope of the line joining end‐systolic pressure and end‐diastolic volume points between children with Still's murmur (M, black) and children without murmur (C, gray). The dotted lines mark the respective end‐systolic volume points. V_0_ representing an end‐diastolic pressure of 0 mmHg is set at 0/0. Children with Still's murmur (M) exhibit a leftward shift of the end‐systolic pressure–volume relationship reflecting an increased LV contractility and a decreased effective arterial elastance.

In children with Still's murmur, the physiologic interaction between the pumping heart and the arterial load seems to be characterized by a leftward shift of the end‐systolic pressure–volume loop reflecting an increased LV contractility and a decreased effective arterial elastance (Fig. 2). One could hypothesize that the analysis of such a hypothetically catheter‐derived pressure–volume loop (Chantler et al. 2008) is likely to reveal a higher change in pressure over change in time and a concomitant decrease in the area representing the potential energy.

Thus, the results of our analysis of the hemodynamic ventriculo‐arterial interaction, while existing on a subtle scale, and without obvious effects on hemodynamics, seem to suggest that Still's murmur is generated by subtle alterations in left ventricular performance and arterial load.

When analyzing the proportional anatomical dimensions of the left ventricle and the aortic root, we found that those dimensions as well as heart rate were not different in children with and without Still's murmur. Thus, our study could neither confirm the results of Gardiner and Joffe (1991), who found significantly lower heart rates in children with Still's murmur, nor the results of Van Oort et al. (1994), who found a significantly smaller aortic root diameter. While the results of our analysis do not support the hypothesis that Still's murmur is generated by an anatomic disproportion of the left ventricle and the aortic root, they do confirm the hypothesis of presence of an increased flow volume across the left ventricular outflow tract.

### Arguments raised by the reviewers of this manuscript

A reviewer argued that differences regarding sound conduction through the chest wall were not taken into account in this study. That could be considered an explanation why not all children presented with Still's murmur. This argument is certainly valid as asthenic children may have indeed better audible murmurs. However, in our study population both weight and height did not differ significantly between cases and controls. Our standardized electronic examination protocol includes also the following item: deformity of the chest (precordial bulge, pectus excavatum, and pectus carinatum). This allowed us also to exclude children with chest deformities and to include only children who were healthy.

Another reviewer suggested that the compliance of the left ventricle and elastance of the arterial tree might be related to the physical fitness of the children studied. This is of course a valid argument. We do not know how many of the children with and without murmurs participated in regular competitive sport programs. Actually, the study population comprised children of relatively young age with a mean age of cases of 5.1 years and a mean age of controls of 5.8 years. Children of this young age participate in such sport programs less often.

### Some limitations of the present study

First, there are no echocardiographic reference values for the VAC ratio in children. In adults, a resting VAC ratio in the range of 0.6–1.2 as determined by echocardiography is thought to represent the “optimal” VAC indicating a near optimal balance between mechanical efficacy and energetic efficiency (Chantler and Lakatta 2012). Actually, the calculated VAC ratios in the present study were distinctly lower so that further pediatric studies are needed to confirm those values and to give new insight into cardiovascular performance throughout childhood (Sunagawa 1983). In view of existing studies attributing a change in arterial elastance to the development of hypertension (Chantler and Lakatta 2012), describing a functional mismatch of E_A_ and E_LV_ in heart failure in aging patients (Kelly et al. 1992), and confirming the prognostic implications of E_A_/E_LV_ in adult chronic heart failure patients (Burkhoff 2013; Ky et al. 2013) such pediatric studies may help to define risk factors for cardiovascular disease later in life. And with regard to Still's murmur, we think that the VAC model and the modulating effects of the arterial system on left ventricular performance (Sunagawa 1983) may also contribute to explain the change in the intensity of the murmur with alteration of position.

Second, we are aware that the use of the formula by Teichholz et al. (1976) for the calculation of left ventricular volumes, albeit applied in children with normal hearts may prompt critical remarks. M‐mode echocardiography was performed under 2D guidance and with perpendicular alignment of the M‐mode beam to the septum, but we are aware that the left ventricular minor axis dimension could have been overestimated as compared with 2D measurements (Feigenbaum et al. 2005). We do not think that the key result of our study was influenced by that methodological approach, as it was applied equally to both cases and controls. However, in further studies on the VAC ratio and its respective normal values in children, we will base calculations also on the biplane Simpson's method. This method has been recommended by the American Society of Echocardiography (Schiller et al. 1979; Lang et al. 2006) and there are studies validating its accuracy and reproducibility in children (Sluysmans and Colan 2009; Cantinotti et al. 2012).

Finally, we would like to mention what should also be considered when discussing the validity of our findings.

A clear strength of the present study lies in the generation of both the clinical and the echocardiographic data using standardized examination and reporting forms and also in the clinical and echocardiographic experience of the pediatric cardiologists who performed the examinations.

Considering the fact that very few healthy children without a murmur undergo cardiac evaluation by echocardiography and taking into account the high prevalence of innocent heart murmurs in children recruitment of controls was indeed difficult. Controls ultimately chosen were asymptomatic children referred because of suspected but finally not confirmed arrhythmia (extrabeats, sinus arrhythmia but no tachyarrhythmias) and children referred because of a cardiac disease in the family history who had a normal ECG and a normal cardiac anatomy with normal M‐mode, 2D, and Doppler findings within the heart and the great arteries.

Another clear strength of the study was the study design because any viable study focusing on Still's murmur must include a control group of healthy children without murmur. When we planned our study we first thought to standardize all M‐mode measurements by expressing them in *z*‐scores. This would have been a mistake. Reference values and *z*‐scores (Kampmann et al. 2000; Sluysmans and Colan 2009) in pediatric echocardiography are based on data from populations of healthy children that include also children with an innocent murmur. To avoid such a loop of “self‐reference” the *z*‐score‐based analysis had to be replaced by the analysis of the proportional dimensions as described.

In conclusion, our study is the first one that used the hemodynamic model of ventriculo‐arterial coupling to contribute to the explanation of Still's murmur. When investigating 2‐ to 10‐year‐old children, we found a significantly lower VAC ratio in children with Still's murmur than in healthy controls. The finding of a significantly lower VAC ratio was even more pronounced in the subgroup of children between 2 and 6 years which is in line with the peak prevalence of Still's murmur in children aged 3–6 years. Our results point out the possibility that a combination of a lower arterial elastance, a higher left ventricular contractility, a significantly larger ejection fraction, and equally larger stroke volume per kg bodyweight may finally find its expression in the typical low‐pitched musical systolic murmur at the lower left sternal border.

Still's murmur is a common clinical finding in everyday pediatrics (Biancaniello 2005). The murmur is not only the most common reason to refer a child to echocardiography but also a cause of considerable doctoral uncertainty and parental anxiety.

Our study provides a physiologic explanation of the phenomenon of Still's murmur. If this physiologic explanation becomes common knowledge among pediatricians and pediatric cardiologists, it is very likely that awareness of the origin of Still's murmur will also reduce doctoral uncertainty. Whether that awareness will also influence the inclination of doctors to refer their patients for an echocardiography remains to be seen.

Referrals are not only driven by doctoral uncertainty but quite frequently by parental anxiety. Our findings enable doctors to give a clear explanation to parents about the origin of Still's murmur. In addition to the assurance that the murmur is “innocent” (Biancaniello 2004) and that the child's heart is normal, doctors can now explain to parents that “the interplay between the heart and the aorta” is just “more lively” in their child, thereby generating a typical musical murmur.

Enabling doctors to communicate this information to parents in the way described above, thereby reducing parental anxiety could be the most important outcome of this study.

## Acknowledgments

The authors would like to express their gratitude to the nurses of the Outpatient Unit of the Division of Pediatric Cardiology, Medical University of Vienna for their dedicated and kind support in the care of all visiting families and the children whose data are reported in the study.

## Conflicts of Interest

None declared.
